# Introduction to Special Issue “Leaders in Cardiovascular Research, Dedicated to the Memory of Professor Adriana Gittenberger-de Groot”

**DOI:** 10.3390/jcdd9040092

**Published:** 2022-03-23

**Authors:** Edi Gittenberger, Robert E. Poelmann, Monique R. M. Jongbloed

**Affiliations:** 1 Naturalis Biodiversity Center, 2333 CR Leiden, The Netherlands; egittenberger@yahoo.com; 2 Institute of Biology, University of Leiden, 2333 BE Leiden, The Netherlands; r.e.poelmann@lumc.nl; 3 Department of Cardiology, Leiden University Medical Center, 2333 ZA Leiden, The Netherlands; 4 Department of Anatomy & Embryology, Leiden University Medical Center, 2333 ZA Leiden, The Netherlands

**Keywords:** cardiovascular development, outflow tract, endocardial cushions, semilunar valves, bicuspid aortic valve, neural crest, aortic wall, coronary arteries, cardiovascular pathogenesis, arterial duct

## Abstract

This Introduction provides both a short reflection on the scientific career of Adriana Gittenberger-de Groot and an overview of the papers that form the basis of this Special Issue giving them a proper perspective. The papers have as a central focus the outflow tract, and include contributions on development and pathology of the ventricles including AV valves, as well as developmental and pathomorphological aspects of the great arteries including semilunar valves and coronary arteries.

## 1. Introduction: Marriage, Medicine and Malacology, Three in One [E.G.]

It was really a surprise, the Festschrift of the Netherlands Malacological Society to commemorate my 65th birthday in 2008. Even more so was the first contribution, written by Adriana C. Gittenberger—de Groot: ‘Marriage and malacology, an adventurous combination’. Adri described the initial 40 years of our living together. She explained why she decided to aim at a career in medical research instead of a clinical specialisation with more restrictions for travelling. She enjoyed our adventurous snail hunting expeditions to the Alps, the Balkans and eventually the Himalaya. After decades of optimal togetherness, life has changed dramatically. Hesitantly, I have accepted the invitation of the editors of this journal to write some introductory sentences. I should have asked her opinion in advance.

**Figure 1:**
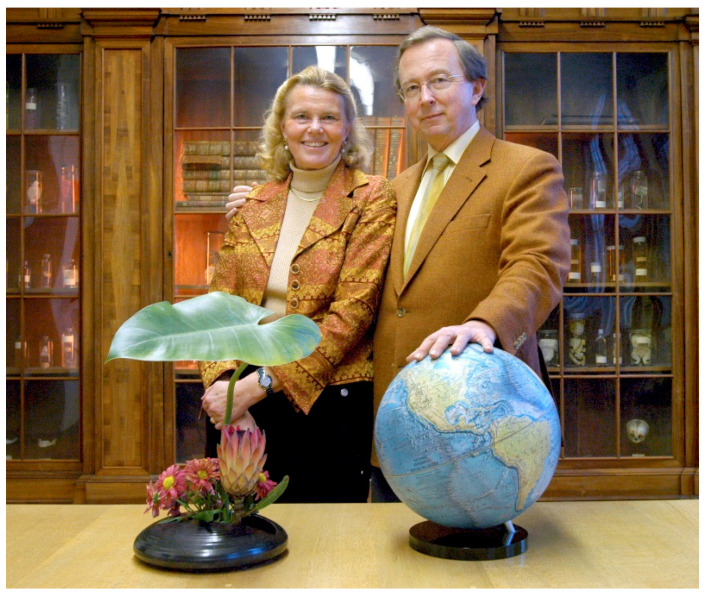
“The Gittenbergers ruling the globe”. Prof. Dr. Adriana C. Gittenberger-de Groot with her husband Prof. Dr. Edi Gittenberger.

Adri was an enthusiastic, tenacious scientist, who believed in serendipity where I noticed her natural talent. Prostaglandin brought her the first breakthrough in research, what resulted in our first visit to the USA. Over the years I learned that the ductus arteriosus is more than a remarkable vessel with programmed arteriosclerosis. Adri was an excellent teacher, who loved to lecture. When we went out to a restaurant for diner after work, she often used the serviette inappropriately to illustrate her most recent ideas about subjects such as the ‘pulmonary push’. “Can you still follow me?”, she could ask after a while, recognizing the near-invisible negative muscle contractions in my face, “no, I see, I’m sorry, but may I nevertheless go on?” And she continued while I waved the waiter away. Over the years her work had become too complicated for me to follow in detail, in particular when anatomy and histology were complemented by gene expression patterns. Our discussions often moved away from the world (Figure 1) of science to the world of scientists, medical scientists in particular.

Medical doctors should decide what kind of pills should be described for the patient, medical scientists should decide what alternatives are still possible without neglecting the publications of competitive working groups. Adri studied the literature irrespective of the authors. She wanted to know how heart and vessels develop in the first place. She considered a dogmatic nomenclature of everything a secondary problem and occasionally even a hindrance for a better understanding. Only rarely she entered the semantic boxing ring that some colleagues seemed to enjoy.

Only once, our scientific ways converged in a joint paper, about Nodal, a chirality-related gene both relevant in heart development and in coiling direction of snail shells [1]. When Nature brought the same message a few years later, we were both annoyed, since that journal had considered our manuscript ‘too specialistic’.

Being out in the field, walking, looking, listening, Adri enjoyed it very much. Despite her vertigo, she eventually loved the mountains, even after a frightening experience during our very first hiking tour in Austria, when we had to use a short iron cable and a short metal ladder to reach the mountain cabin. Bird-watching became one of our favourite hobbies. Adri understood the remarkable aberration of biologists such as me, people that may acquire a feeling of happy excitement after a vague view of a bird that might be, yes, indeed (!), the very rare yellow-tailed warbler according to the field guide. While peering at it, she joined in the discussion about dots or stripes.

**Figure 2:**
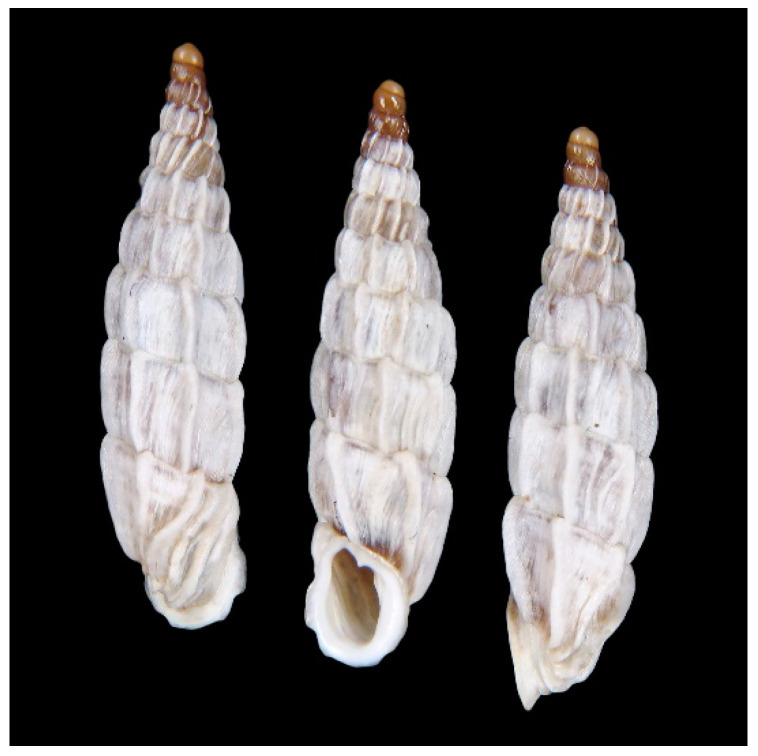
*Albinaria adrianae.* Courtesy of Guido T. Poppe and Philippe Poppe.

Adri enjoyed snail hunting, in particular the inhabitants of rock-faces and, indeed, she discovered the beautiful Greek species that I described as Albinaria adrianae (Figure 2).

When our youngest son learned scuba-diving, she followed him and urged me to do the same. In vain I referred to the evolutionary origin of terrestrial life and the unnatural behaviour of swimming. She stayed firm and arranged lessons how to swim first and scuba diving shortly afterwards. A full and wonderful dimension was added to our lives. Whenever possible we combined our journeys, holidays, conferences, with scuba diving. That is why we were in Bonaire early in 2020. An unforgettable recent experience were six joint excursions to the Kingdom of Bhutan, where I was invited to start an inventory of the molluscan fauna. Adri was immediately enthusiastic. She appreciated the atmosphere, the people, nature, the blooming rhododendrons high in the Himalaya. Never ever she protested when we had to adapt to non-European circumstances, on the contrary. Back in the capital we indulged in a hamburger with pommes frites, always together.

“The future is bright.” was her last sentence in the Festschrift in 2008. She was right. After that year, 12 wonderful years followed. A few more, she had hoped, grateful for the past.

## 2. The Special Issue: The (Bio)medical Legacy of Adriana Gittenberger-de Groot

In the JCDD issue “Leaders in Cardiovascular Research, a Special Issue dedicated to the memory of Professor Adriana Gittenberger-de Groot”, committed to Adriana’s extensive career, we see a reflection of the versatility of subjects that have been explored by dedicated researchers, driven by the desire to ultimately solve part of the puzzle that encompasses the mechanism of development of structural heart disease, affecting so many patients worldwide [2]. Many mismatches in cardiovascular development particularly result in ventricular septal defects and in outflow tract malformations, that showed a continued presence in Adriana’s work. Furthermore, late onset of specific disease processes such as aneurysm of the ascending aorta associated with bicuspid aortic valve [3], might find their origin during embryonic development justifying the inclusion in the category “congenital heart disease”.

**Figure 3:**
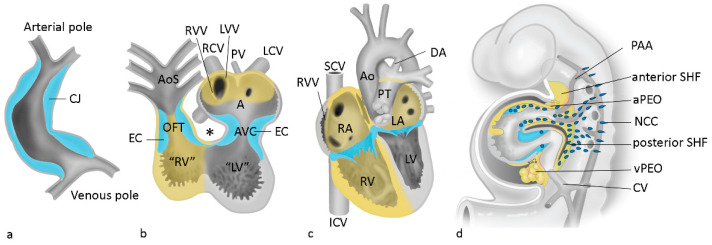
Cardiac development: Contributions from the second heart field and neural crest cells. Panels (**a**–**c**) reflect successive stages of development. Panel (**d**) represents a lateral view showing the migration path of neural crest cells (NCC). A: common atrium, Ao: aorta, AoS: aortic sac, AVC: atrioventricular canal, CJ: cardiac jelly, CV: cardinal vein, DA: ductus arteriosus, EC: endocardial cushions, ICV: inferior vena cava, LA: left atrium, LCV: left cardinal vein, LV: left ventricle, LVV: left venous valve, NCC: neural crest cells, OFT: outflow tract, PAA: pharyngeal arch artery, PEO: pro-epicardial organ, PT: pulmonary trunk, PV: pulmonary vein, RA: right atrium, RCV: right cardinal vein, RV: right ventricle, RVV: right venous valve, SCV: superior vena cava, SHF: second heart field. * inner curvature of the looping heart. Derived with permission from Gittenberger-de Groot et al. [4].

The majority of congenital cardiac malformations involve ventricular and outflow tract septation with the participation of many interacting cellular players (Figure 3) such as the first/second heart field, neural crest, myocardium, endocardium, epicardium and endoderm. Concentrating on ventricles and outflow tract in development, molecular genetics, pathology and patient management, all these aspects can be encountered in this Special Issue. Several groups of papers can be discerned, dealing with cardiac (mal)development as well as with the aortic valve and the main arteries such as aorta, coronary arterial branches and ductus arteriosus.

*OFT and ventricular myocardium—developmental, molecular and genetic aspects.* A review paper by Sendra et al. [5] discusses the complexity of heart progenitor cells important to understand congenital heart defects. Three key aspects in early development are important, dealing with 1. the segregation of endocardial and first and second heart field myocardial lineages, 2. the signalling cues that drive differentiation and 3. the transcriptional heterogeneity of cardiomyocyte progenitors. Novel approaches such as single cell transcriptomics (see also Peterson et al. [6]), lineage tracing (see also Stefanovic et al. [7] and Johnson et al. [8] and epigenomics (see also Grunert et al. [9], live imaging and functional analyses will transform our understanding of congenital anomalies.

The development of new techniques also opened avenues for more in-depth investigation of signalling and inducing factors involved in the development of congenital and structural heart disease and their roles at other levels of interactions. Cardiac lineage tracing is further explored by Peterson et al. [6]. They show the value of chicken-quail chimeras [10] and retroviral labelling [11]. Furthermore, they elaborate on genetic lineage tracing markers for first and second heart field, myocardium, neural crest, epicardium, and endothelium. Finally, DNA barcoding allows for complex labelling methods incorporating also CRISPR-Cas9 based approaches. The cellular contributions to the conotruncal area are reviewed by Stefanovic et al. [7]. These authors take into account the many cell types responsible for proper heart formation and the consequences of deregulation of transcription factors such as Isl1 and FGF10 for myocardial cells, BMP and T-box factors for endocardium, and Wnt1 and FGF8 for cardiac neural crest cells. Cardiac anomalies are known to be related to complex signalling defects including retinoic acid, Hox transcription factors or chromosomal syndromes. The transcription factor AP-2α (Johnson et al. [8]) is expressed in several tissues during development including pharyngeal ectoderm and cardiac neural crest. Deficient mice present with complex anomalies such as double outlet right ventricle (DORV) and transposition of the great arteries (TGA).

At the molecular and genetic level many cascades have been investigated. The TGFbeta constitute an important family of regulating factors. Chakrabarti et al. [12] relay the requirement of TGFβ3 for cardiovascular development as investigated in a mouse model. The diverse phenotypes were present in about two thirds of null mice, including (right) ventricular myocardium, OFT septal and alignment defects, abnormal aortic and pulmonary walls and thickened semilunar or AV valves. In vitro and biochemical studies indicated that TGFβ3 was required for collagen matrix reorganization involving SMAD and MAP-kinase dependent pathways.

Leung et al. [13] investigated the cardiomyocyte specific role of the small GTPase Rac1 in the ventricular wall in Nkx2.5 transgenic mice. Rac1 deficiency impairs cardiomyocyte elongation and organization as well as proliferative growth, implicating a role of Rac1 in OFT alignment and compaction of the myocardium.

From a genetic point of view, Grunert et al. [9] correlated genetic and epigenetic differences in Tetralogy of Fallot, studying monozygotic twins. They performed genome-wide high-throughput sequencing of twin pairs with TOF. DNA methylation changes in regulatory regions of cardiac-relevant genes were demonstrated.

*The cardiac valves—developmental, pathomorphological and clinical aspects.* Epicardial derived ells (EPDCs) are relevant for pathogenesis of myxomatous valve disease, as was demonstrated by Wolters et al. [14] who reviewed critical events in the development of the atrioventricular junction, including the role of the epicardium in the development of the AV valves. The parietal AV cushions receive a contribution from epicardium-derived cells (EPDCs) in concordance with results indicated by Gittenberger-de Groot et al. in a chick model [15].

Poelmann et al. [16] described endocardial cushion formation in crocodiles and their role in semilunar valve development. Reptiles in general end up with two aortas besides the pulmonary trunk, while all three main trunks show a bicuspid semilunar valve. It is postulated that “missing the third leaflet” might be related to lack of neural crest or epicardial derived cells.

Zwanenburg et al. [17] searched for predictors of adverse human left ventricular development caused by fetal aortic stenosis using fetal ultrasound. Speckle-tracking recordings were performed to assess myocardial strain. After pregnancy termination. The degree of reduction in myocardial deformation corresponded to the amount of pathological endocardial fibrosis, indicating myocardial deformation on fetal ultrasound as a potential marker for left ventricular structural damage.

Kruithof et al. [18] investigated semilunar aortic valve disease, both regurgitant and stenotic. They demonstrated the occurrence of superimposed tissue in human samples. The regurgitant valve showed this in the free edge, leading to a thicker valve, while the stenotic valve showed this on the aortic face. These highly active areas are populated by myofibroblasts within a varied extracellular matrix that is collagen-rich in the stenotic valve. Çelik et al. [19] studied a patient cohort with bicuspid semilunar aorta valve associated with aortopathy leading to dilation, aneurysm and eventually dissection. Prevalence, predictors and outcome (1–20 years) of 48 patients were analysed. Grewal et al. [20] described earlier the association of BAV with aortopathy as a developmental maturation defect of the aortic root. A second contribution to this Issue by Çelik and colleagues [21] described the impact of intervention in severe but asymptomatic aortic stenosis by valve replacement and long-term survival. Survival rates after valve replacement surgery were significantly higher after 1–10 years follow-up.

*Thoracic aorta—histopathomorphological and (patho)developmental) aspects.* Grewal et al. [22] reported on the histological structure of the aortic wall in patients with an acute type A aortic dissection (TAAD) showing elastin pathology, mucoid accumulation, and smooth muscle cell nucleic loss in overall medial degeneration while also associated with a thinner intimal layer. The authors hypothesize a developmental defect caused by a multiple hit including a tear in the intima, a diseased media and progression towards the vasa vasorum.

Thiene et al. [23] reviewed the aorta as an organ that deteriorates with age. They investigated many types of pathology in humans and brought also the possibility of an allograft approach in sheep. The aorta with its lamellar units consists of elastic fibres in a collagen-rich extracellular matrix interspersed with smooth muscle cells. Pathology may require replacement sometimes including failing valves. There may be congenital defects such as bicuspid semilunar valve and isthmus coarctation, genetic defects such as Marfan and William syndromes, degenerative diseases and trauma. Antigenicity may prevent the replacement of a sickened aorta using homografts from donors. After proper decellularization and implantation in sheep, endogenous cell repopulation was shown in both the valve and aortic wall without structural deterioration.

Another defect of the aorta relates to the right-sided aortic arch, an early developmental defect in remodelling of the pharyngeal arch arteries [24]. Van Rosendael et al. [25] relay a malformation found in adult patients associated with the branchpoint of an aberrant left subclavian artery (arteria lusoria) from the right-sided aortic arch. In this configuration a Kommerell’s diverticulum and persisting ductus arteriosus form a vascular ring which may result in (symptomatic) esophageal or tracheal compression. Serial follow-up is warranted in adult patients with Kommerell’s diverticulum with small size and no symptoms, eventually leading to surgical intervention in patient who become symptomatic or show significant increase in diameter of the diverticulum.

Coronary arteries—The Leiden Convention Coronary Coding System and late follow up after surgery for transposition of the great arteries. Another highlight of Gittenberger-de Groot’s research comprised the early formation of the coronary vasculature [26] and the branching pattern of the coronary arteries (Gittenberger-de Groot et al. 2018 [27]). In this Special Issue cardiovascular thoracic surgeon Prof. Dr. Mark Hazekamp [28] stresses the value of an unequivocal and simple coronary coding system developed by Adriana, called the Leiden Convention. This can be used in hearts with congenital heart disease (CHD), such as transposition of the great arteries (TGA), independent of their relative anatomical position which can vary in different forms of congenital heart disease. A recent adjustment of this system to facilitate its use in imaging modalities, facilitates communication between paediatric cardiologists and paediatric heart surgeons [29]. This is further explored by Katekaru-Tokeshi et al. [30] studying patients with a single coronary artery. The Lipton classification (not intended for structural heart diseases) has also been applied but in complex CHD the Leiden Convention is better applicable. In pulmonary atresia the use of both systems is limited.

Due to advancements in surgical and interventional treatments, an increasing number of patients with CHD reaches the adult age. Engele et al. [31] systematically reviewed the follow-up into adulthood of a patient population with TGA that received an arterial switch operation with coronary transfer in early life. Anatomical high-risk features found by coronary computed tomography included stenosis, acute angulation, kinking and an interarterial course. An individualized coronary follow-up strategy is advisable, at least until significant duration of follow-up is available.

*Arterial duct.* During her life, Adriana studied the influence of prostaglandins on the ductal integrity as clinical reports brought devastating effects on the survival rate of treated neonates [32]. Following her initial reports the pharmaceutical industry lowered the recommended dose promptly, resulting in the expected positive outcome. In addition, later on, the arterial duct kept her interest [33,34].

In the current special issue, Saito et al. [35] performed transcriptome analysis of the arterial duct and demonstrate differential gene expression patterns between the closing and the patent ductus arteriosus, building on the earliest research projects of Adriana Gittenberger-de Groot [36]. The patent ductus exhibited aorta-like elastic lamellae and a poorly developed intimal thickening. Neural crest-related genes including JAG1 and the protein calponin were highly expressed in the tunica intima and media of the closing ductus but not in the patent one. Second-heart field related genes such as ISL1 on the other hand were enriched in the patent ductus sample indicating participation of another cell lineage. Fedrigo et al. [37] reported another clinical phenomenon of the developing ductus, being premature closure causing sudden intrauterine death. Abnormal ductal remodelling showed lack of intimal cushions, cystic medial necrosis and smooth muscle cell apoptosis as pathological substrates.

**Figure 4:**
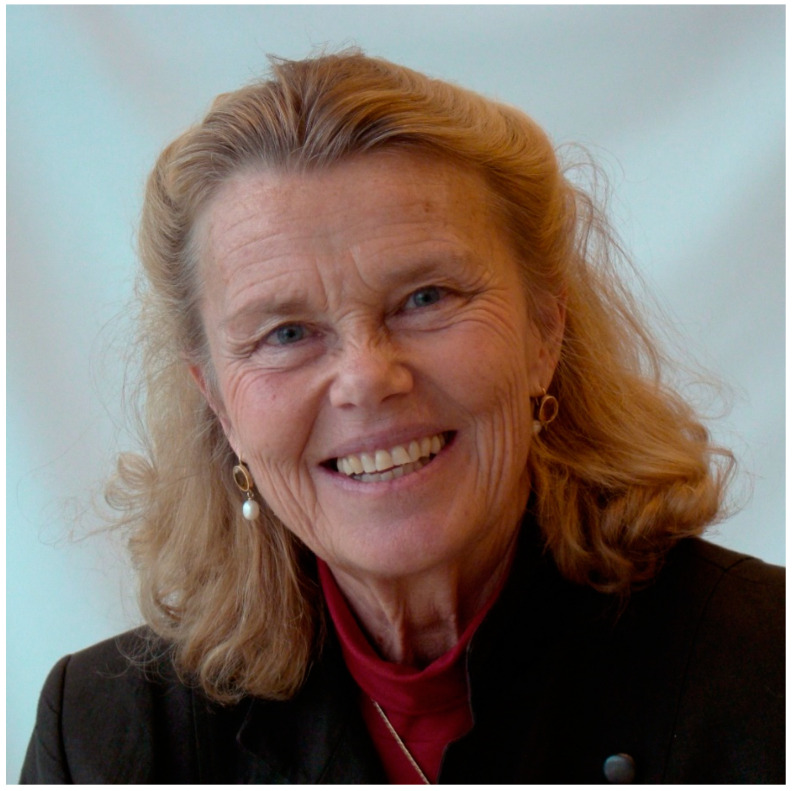
Prof. Dr. Adriana C. Gittenberger—de Groot. 1945–2020.

In conclusion the legacy of Adriana Gittenberger-de Groot (Figure 4) combines the importance of proper knowledge of embryonic development not only for explaining congenital cardiac malformations, but also for late onset clinical issues such as the onset of arteriosclerosis and the repair of myocardial infarction. The content of this special issue reflects the versatility of Adriana’s career, during which she worked with remarkable scientists and made many friends, many of whom made a contribution to this special issue. Her creativity, combined with her intellectual spirit and passion for research allowed the development of a myriad of new concepts, that will be the driving force for many more exciting studies of cardiovascular development. We will cherish our memories and her legacy dearly.
